# Rio Doce Acoustic Surveys of Fish Biomass and Aquatic Habitat

**DOI:** 10.1002/ieam.4285

**Published:** 2020-06-18

**Authors:** Dustin Hobbs, Marie Bigot, Ross Edward William Smith

**Affiliations:** ^1^ Hydrobiology Brisbane Queensland Australia

**Keywords:** Hydroacoustics, Sonar, Biomass, Recovery, Rio Doce

## Abstract

Following the failure of the Fundão mine tailings dam in Brazil, approximately 32 million cubic meters of Fe ore tailings were released into the downstream riverine system. The postevent monitoring surveys implemented the use of noninvasive acoustic methods to improve the understanding of the fish biomass distribution patterns and aquatic habitat condition of the impacted reaches of the Rio Gualaxo do Norte, Rio do Carmo, and Rio Doce. The primary focus of the program was to collect hydroacoustic measurements of fish biomass, which was accompanied by sonar imaging of aquatic habitats using high‐resolution side scan and downward imaging sonar at each site visited. The biannual surveys began in April 2017 and were fundamentally a multiple control–impact design because before data (prior to the dam failure event) were not available. Up to 22 sites were visited for each survey, including reservoir and river sites. Results indicate similar levels of instream habitat between control and impact river and reservoir sites. Average volumetric biomass was not significantly different between impact sites and their corresponding controls in the August 2018 survey (latest to date). Preliminary temporal analysis of the biomass data set collected indicates that either stable or increasing levels of biomass are detected at tailings impacted sites within the Rio Doce. *Integr Environ Assess Manag* 2020;16:615–621. © 2020 Hydrobiology QLD Pty Ltd. *Integrated Environmental Assessment and Management* published by Wiley Periodicals LLC on behalf of Society of Environmental Toxicology & Chemistry (SETAC)

## KEY POINTS

- We monitored the recovery of fish biomass and benthic habitat of the Rio Doce, Brazil, catchment after the Fundão mine tailings dam rupture using noninvasive acoustic techniques.
- Results to date show no discernible differences between appropriate control and impact sites, and temporal trends in biomass are either holding steady or improving at sites that were in the tailings flow path.

## RESUMO

Após o rompimento da barragem de rejeitos da mina do Fundão no Brasil, cerca de 32 milhões de metros cúbicos de rejeitos de minério de Fe foram libertados no sistema fluvial a jusante. Monitoramentos pós‐evento foram implementados com a utilização de métodos acústicos não invasivos visando ampliar a compreensão dos padrões de distribuição da biomassa de peixes e condições dos habitat aquáticos em trechos impactados do Rio Gualaxo do Norte, Rio do Carmo e Rio Doce. A aquisição de medições hidroacústicas sobre a biomassa de peixes foi o foco principal do programa, seguido pela obtenção de imagens dos habitats aquáticos através da utilização de um sonar de varredura lateral de alta resolução e um sonar de imagem vertical, em cada local visitado. Os levantamentos de campo semestrais tiveram início em Abril de 2017 e seu design amostral foi baseados na concepção “trechos controle – trechos impactados” uma vez que não existem dados disponíveis para o período anterior ao evento (rompimento da barragem). Em cada levantamento foram visitados até 22 pontos de amostragem, incluindo vários reservatórios e rios. Os resultados indicaram níveis de habitat similares entre os pontos controle e impacto, de rios e reservatórios. No monitoramento mais recente (Agosto/2018) a biomassa volumétrica média não foi significativamente diferente entre os locais de impacto e os seus correspondentes pontos controles. A análise temporal preliminar do conjunto de dados sobre a biomassa indicou níveis estáveis ou crescentes deste parâmetro nos locais impactados pelos rejeitos, no Rio Doce. *Integr Environ Assess Manag* 2020;16:615–621. © 2020 Hydrobiology QLD Pty Ltd. *Integrated Environmental Assessment and Management* published by Wiley Periodicals LLC on behalf of Society of Environmental Toxicology & Chemistry (SETAC)

## INTRODUCTION

The failure of the Fundão mine tailings dam in the upper reaches of the Rio Doce catchment (Minas Gerais, Brazil) on 5 November 2015 led to the release of the approximately 32 million cubic meters of Fe ore tailings into the Rio Gualaxo do Norte and downstream to the Rio do Carmo and Rio Doce. The tailings material reached the marine environment on 22 November 2015 after travelling along approximately 680 km of river. A large portion of the mine tailings (~27 million m^3^) was deposited within the Candonga Reservoir (Risoleta Neves hydroelectric plant) approximately 100 km downstream of the Fundão mine tailings dam.

The initial plume of sediment and continued residual turbidity within the impacted river system are known to have significantly affected fish populations and available aquatic habitat (Fernandes et al. 2016; Golder Associates 2016). There were reports of fish deaths associated with the initial plume. Some of the impacts to the fishes of the lower Rio Doce, below the Aimores reservoir, are also attributable to low dissolved O water released from the stagnant bottom water of the various reservoirs along the Rio Doce in preparation of the arrival of the tailings plume.

Collection of in‐situ information on the health of the aquatic ecosystem did not start until April 2017 due to delays in agreement with and approval by the regulators of monitoring methodology, permit, and personnel. In order to gain a data set within a more practical timeframe, a rapid deployment of acoustic methods was chosen as a nonimpact methodology, which did not require a fishing permit (no fish were collected or harmed during the surveys). These methods, widely adopted for the purpose of fisheries management, have been applied in many freshwater systems in Brazil (Bezerra‐Neto et al. 2012, 2013) and around the world (Guillard et al. 1994, 2012; Kubecka and Duncan 1998; Matveev 2007; Matveev and Steven 2014). This acoustic survey program was implemented in 2017 and has since been continued to provide an ongoing, complimentary data set to those being collected by other entities.

## METHODOLOGY

The biannual surveys performed to date visited a total of 22 sites. The survey site details including site name, water body (river or reservoir), impact type (control or in the tailings flow path [“impacted”]), and site map are provided in Supplemental Data.

The primary focus of the present program was to collect hydroacoustic measurements of fish biomass using a Biosonics DT_X system with a 6.4° split beam transducer operating at 201 kHz connected to a differential global positioning system (Sokkia DGPS, <1 cm accuracy) for spatial georeferencing. Detailed methods are provided in Supplemental Data. Average volumetric fish biomass (expressed in grams per cubic meter) was estimated for each replicate (minimum of 3) transect performed at each site visited. The transects were run parallel to the shore for approximately 1000 m, where depth and flow allowed (or shorter distances otherwise). Many pelagic to benthopelagic species of fish are known to occur in the Rio Doce (Vieira 2010). The mobile biomass survey methods targeted primarily pelagic fish species; therefore demersal fish species (e.g., various species from the Auchenipteridae, Callichthyidae, Loricariidae, and Pimelodidae families known to inhabit the Rio Doce [Vieira 2010]) were likely undersampled in the present program.

Accompanying sonar measurement of water depth, bottom roughness, and bottom hardness (Humminbird Helix 9, 200 kHz down beam) and imaging of aquatic habitats using high‐resolution side scan and downward imaging sonar (Humminbird Helix 9, 1200 kHz) were also collected at each site.

## FISH BIOMASS RESULTS

The last spatial analysis of biomass available at the time of writing the present manuscript was for the August 2018 survey. In the present survey, 20 sites were visited. After processing of the data collected and removal of transects that were too noisy to process accurately, hydroacoustic measurements of fish biomass were generated for 9 reservoir sites and 8 river sites.

**Figure 1:**
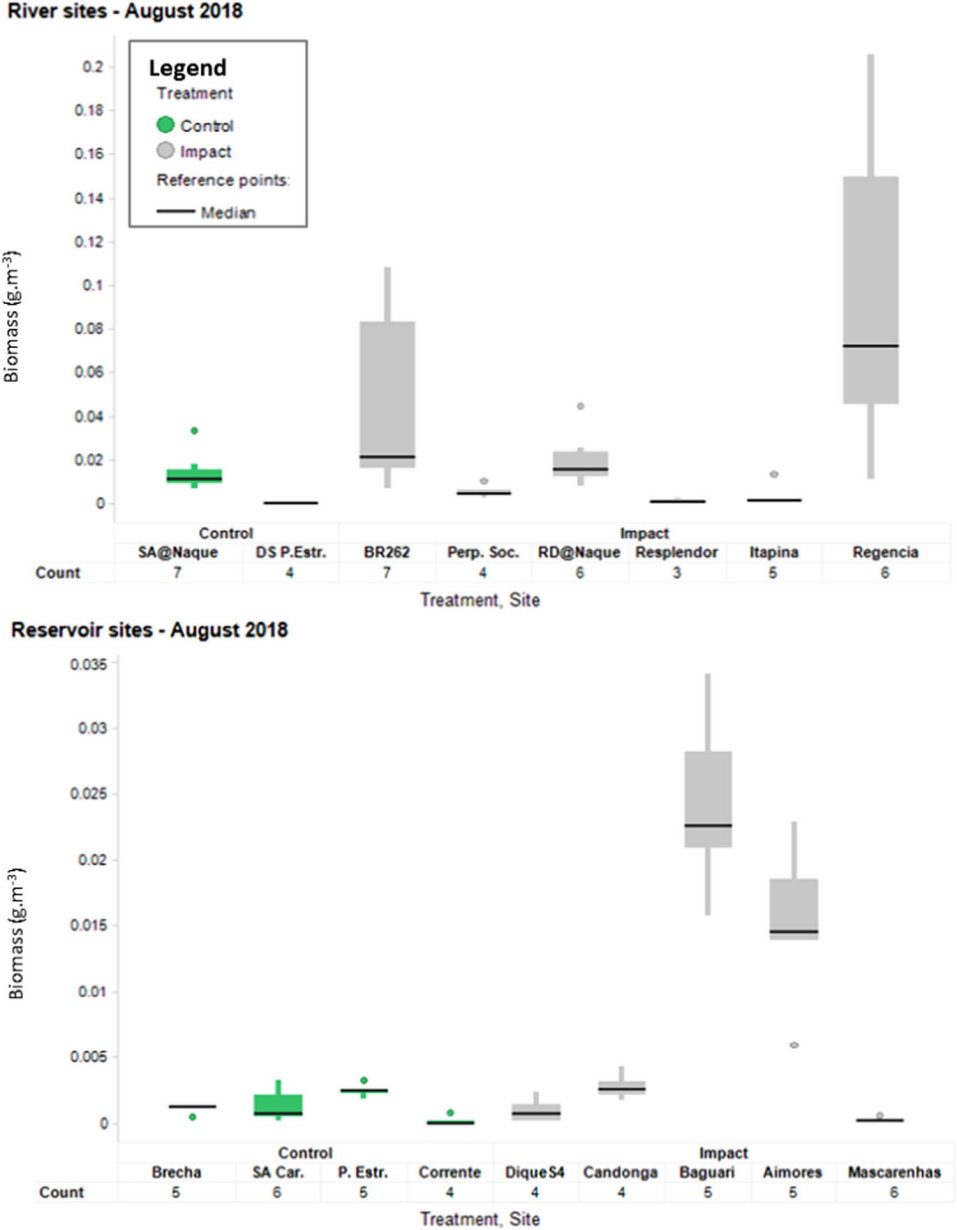
Graphical summary (boxplot) of biomass estimates across river and reservoir sites investigated in August 2018. DS P Estr = Downstream Porto Estrela; Perp Soc = Perpetuo Socorro; RD = Rio Doce; SA = Santo Antonio; SA Car = Sá Carvalho.

Average volumetric biomass estimates for the August 2018 survey are presented in Figure 1. Fish were detected at all impacted sites investigated along the Rio Doce, at variable densities. The variability of estimated biomass levels between sites and treatment (i.e., control and impacted) may be due to a number of factors, including variability of habitat types available, depth of the area surveyed (i.e., likelihood of encounter), time of day at which the measurement was taken, and other parameters. Results of statistical analyses (ANOVA, significance level *p* < 0.05) were as follows (all biomass estimates are reported as average of all transects per site ± standard deviation):

Reservoir sites:

- Fish biomass was significantly higher at the 2 impact sites Baguari (24.3 ± 7.1 mg/m^3^) and Aimores (15.2 ± 6.3 mg/m^3^) compared with all other reservoir sites investigated (0.2 ± 0.4 to 2.8 ± 1.1 mg/m^3^).
- Very few fish were detected on the echograms at Mascarenhas, which was unusual compared with previous surveys. Fish biomass at this site (0.3 ± 0.2 mg/m^3^) was significantly lower compared with two of the control sites (Brecha and UHE Porto Estrela, 1.1 ± 0.3 and 2.5 ± 0.5 mg/m^3^, respectively) and the impacted site Candonga (2.8 ± 1.1 mg/m^3^), located upstream.
- Fish biomass estimates for the most upstream impacted sites Dique S4 (1.0 ± 1.0 mg/m^3^) and Candonga were not significantly different from the relevant control sites investigated (0.2 ± 0.4 to 2.5 ± 0.5 mg/m^3^).
- The control site Corrente displayed very few fish, with no single echo detected on 3 out of 4 replicate transects.

River sites:

- Greater variability of the biomass estimates was observed at river sites, compared with reservoirs.
- For the present survey, Santo Antonio at Naque was the only relevant control site included in the statistical analysis. The data collected at Rio Piranga could not be included as a result of noise and limited depth, providing too little sample for analysis. No fish were detected at the other control site downstream of the Usina Hidreléctrica (UHE; hydroelectric power plant) Porto Estrela.
- Biomass estimates from the Rio Doce (impact site, 20.2 ± 13.3 mg/m^3^) and Santo Antonio (control site, 14.7 ± 9.1 mg/m^3^) at Naque were not significantly different.
- No suitable control site could be found in the lower reach of the Rio Doce to date. Itapina and Resplendor had significantly less biomass (3.8 ± 5.3 and 1.1 ± 1.2 mg/m^3^, respectively) compared with the control site Santo Antonio at Naque. Given that the location of these 2 impacted sites is a few hundred kilometers downstream in the Rio Doce in a much wider channel, it is suspected that the distribution of fish at these lower reach sites differs from the upper reaches as a result of morphological differences in the river attributes. In lowland rivers, studies indicate that fish are likely to occur in lower densities in the areas targeted by the hydroacoustic set‐up used for the Rio Doce program (i.e., middle to deep layers, below 1‐m depth) and will most likely be found in the surface layer and littoral area, in relatively lower numbers during daylight (Kubecka and Duncan 1998; Wolter and Freyhof 2004). Therefore, it is likely that only a minority of the fish present are detected using the methods adopted, mainly due to logistical constraints requiring sampling during daylight. Biomass estimates at Itapina and Resplendor were consistent with previous measurements. Long‐term temporal trend analyses of the data sets will provide a better assessment of the recovery process at these sites.
- Average biomass estimates at BR262 and Regencia (4.8 ± 4.2 and 9.6 ± 7.7 mg/m^3^, respectively) were highly variable. At Regencia, this variability was associated with a number of larger fish observed on some of the replicate transects, therefore reflecting high fish mobility at that site. The biomass estimate at BR262 was not significantly different from that of the control.

**Figure 2:**
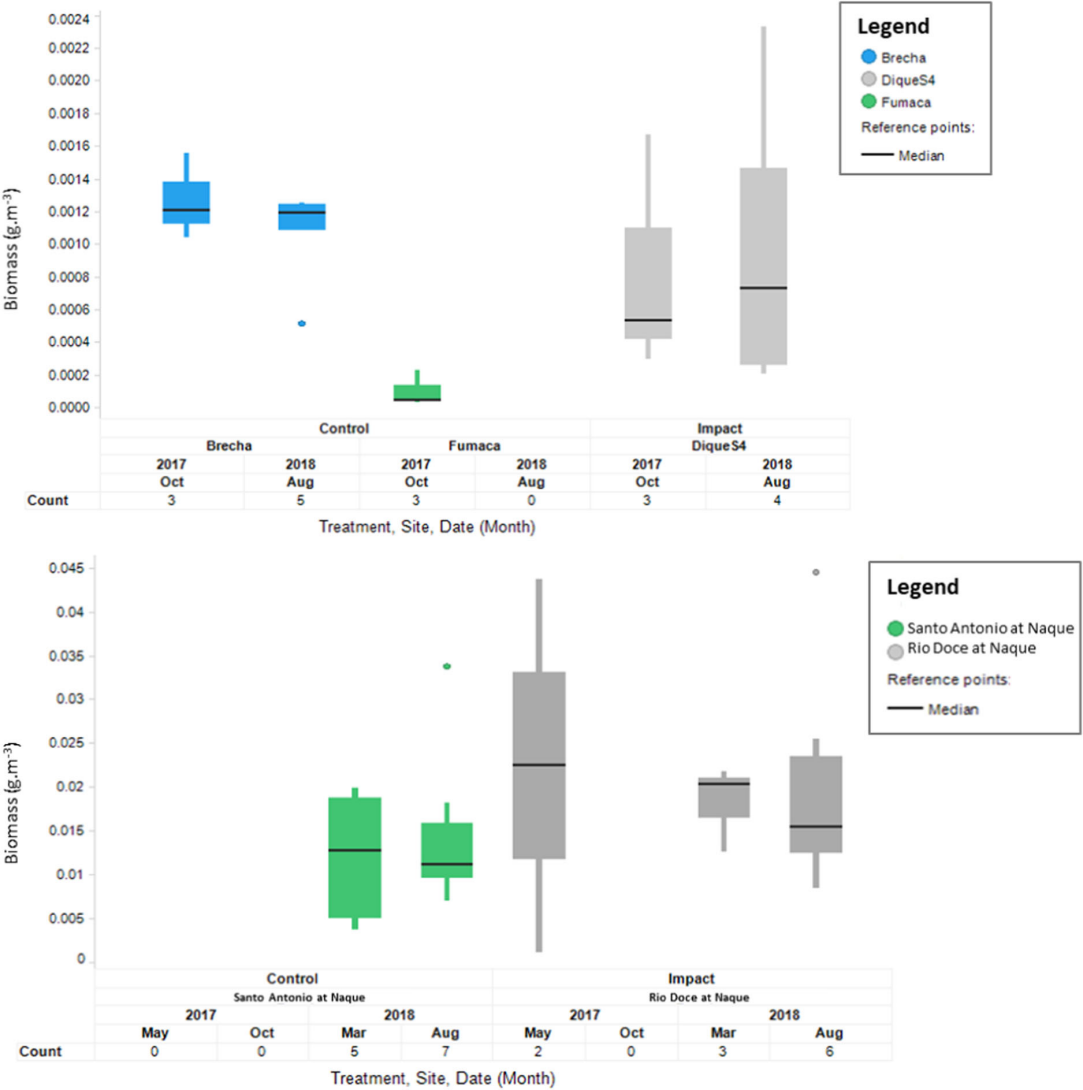
Boxplot of temporal measurements of biomass at corresponding control and impact reservoir sites above Candonga (top graph) and at river sites at Naque (bottom graph).

Preliminary temporal biomass analyses were completed at the end of the August 2018 survey. For the analysis of biomass trends over time, the impacted reaches were evaluated in 3 major sections: Upper Reach, Middle Reach, and Lower Reach. Key findings and observations from this program to date were as follows:

Upper reach (Figure 2)

- Dique S4 was the only impact reservoir site surveyed above Candonga, and it has been sampled only in the late dry season to date.
- Although highly variable, the average fish biomass at Dique S4 (0.8 ± 0.7 and 1.0 ± 1.1 mg/m^3^, in August 2017 and 2018, respectively) was within the range observed at the 2 most upstream reservoir control sites (Fumaca and Brecha, 0.1 ± 0.1 to 1.3 ± 0.3 mg/m^3^). These sites are the 2 most relevant controls available for comparison with Dique S4.

Middle reach (Figure 2)

**Figure 3:**
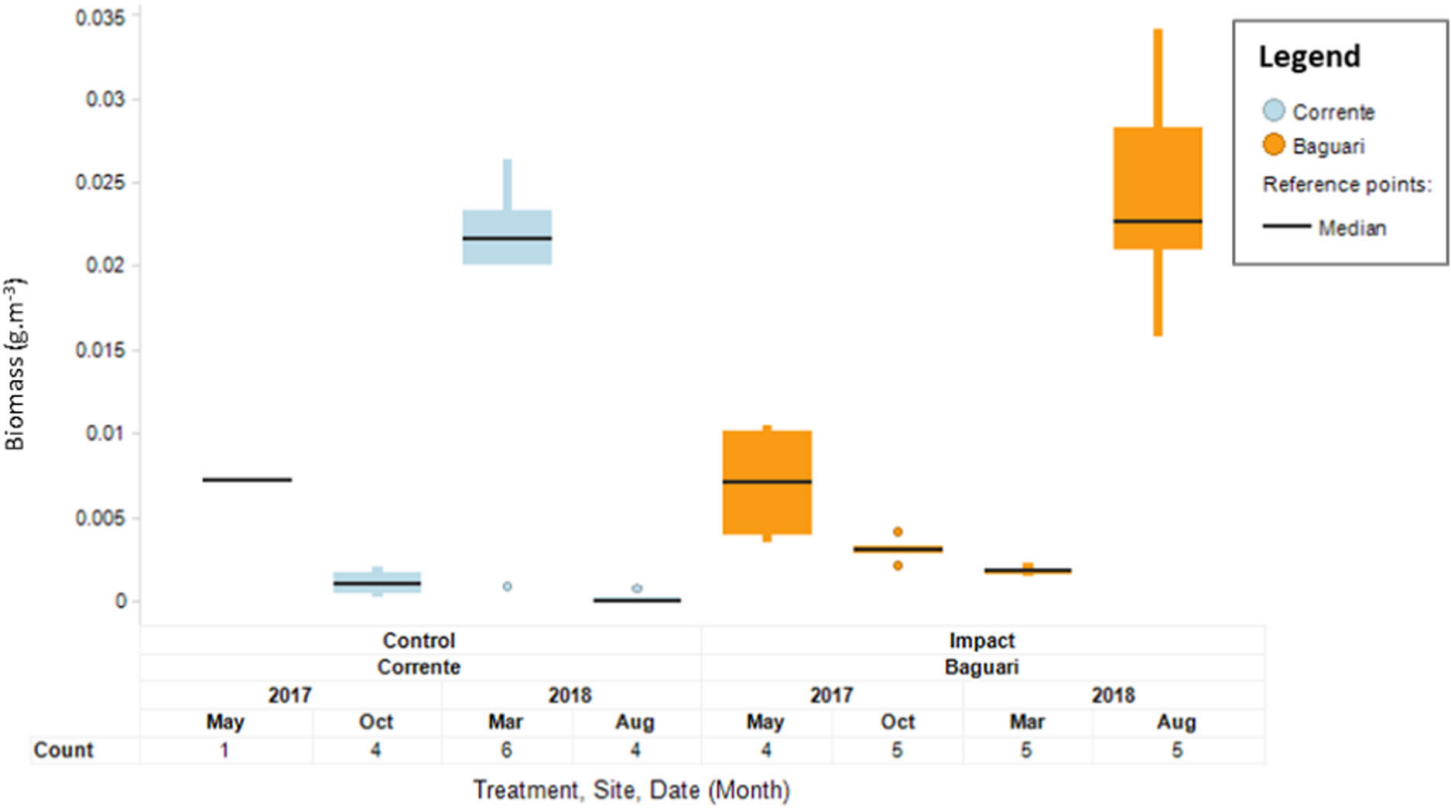
Boxplot of temporal measurements of biomass at corresponding control and impact reservoir sites in the middle reach.

**Figure 4:**
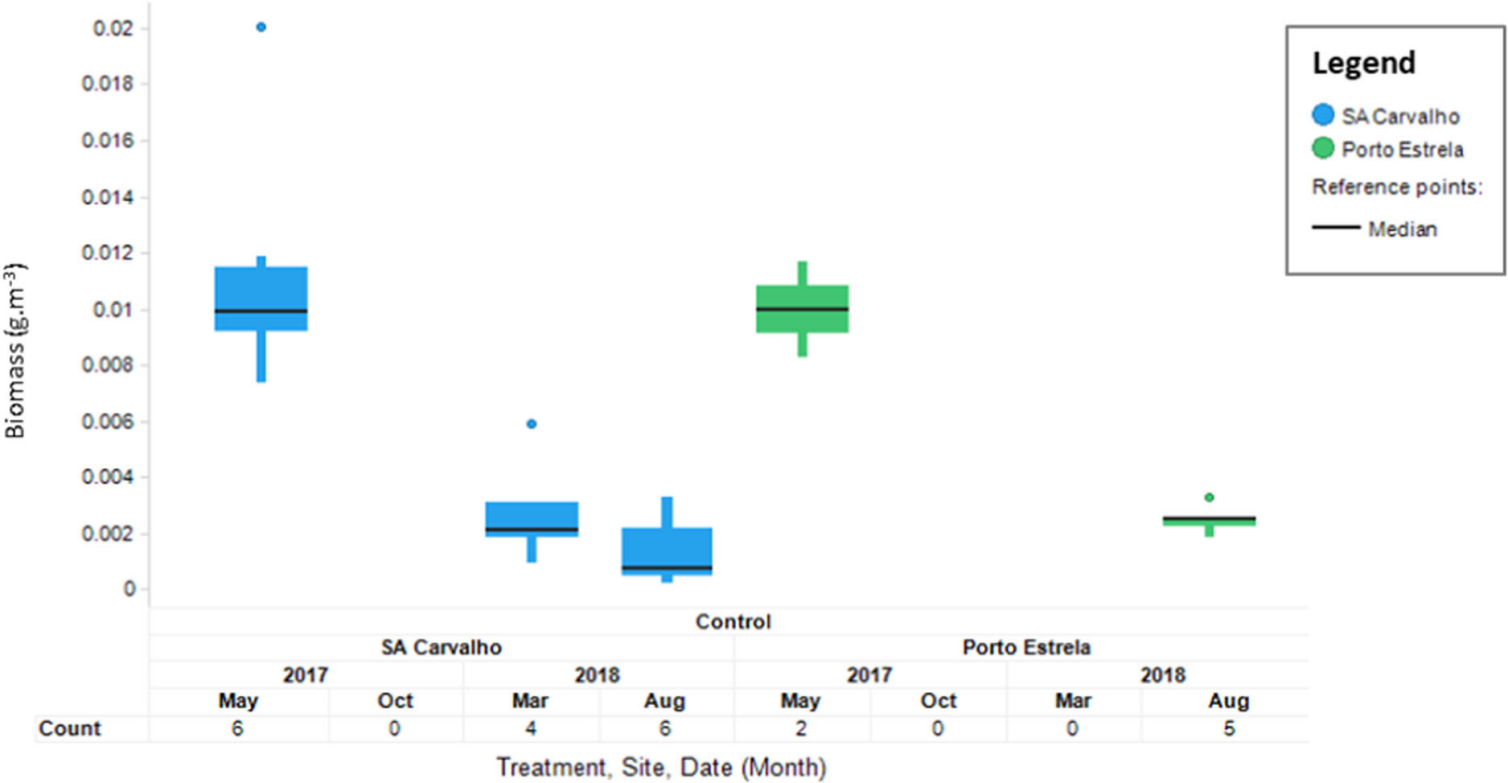
Boxplot of temporal measurements of biomass at control reservoir sites in the middle reach.

- No significant difference in biomass was found between the Rio Doce (impact, 18.2 ± 4.9 to 22.4 ± 30.1 mg/m^3^) and Santo Antonio (control, 12.0 ± 7.5 to 14.7 ± 9.1 mg/m^3^) at Naque river sites in the last 2 surveys (Figure 2, ANCOVA, Treatment, Time, *p* > 0.05). This indicates a consistent similarity between a control and an impact site located in close proximity to each other.
- For the reservoir sites, no significant difference was found (Figure 3, ANCOVA, treatment, time, *p* > 0.05) between biomass results over time collected at Baguari (1.8 ± 0.2 to 24.3 ± 7.1 mg/m^3^) compared with the control Corrente (0.1 ± 0.1 to 19.0 ± 9.1 mg/m^3^), both located in close proximity to each other. In August 2018, the average fish biomass at Baguari was significantly higher compared with Corrente, but the temporal analysis indicated that there was no significant increasing trend at that site through time. Future surveys will confirm this.
- Interestingly, it is noted that the average biomass at the 2 control reservoirs Sá Carvalho and Porto Estrela decreased significantly over time since the initiation of the hydroacoustic program (Figure 4).

Lower reach

- There was no significant increasing or decreasing trend observed to date at Regencia (ANCOVA, *p* > 0.05, Supplemental Data Figure SD1). Greater fish biomass was observed in March 2018 in Resplendor and Itapina compared with other surveys to date at these sites (Supplemental Data Figure SD1). The statistical analysis of biomass density over time (ANCOVA, site, time, season) indicated that seasonality (late wet vs late dry season) was the significant factor (*p* < 0.05) driving biomass variability over time at these sites rather than time through the 2 y of sampling. This will be further assessed and confirmed with the addition of further data.
- No significant temporal trend was observed in reservoirs of the lower Rio Doce (Aimores and Mascarenhas, Supplemental Data Figure SD2).

## AQUATIC HABITAT RESULTS

Transects were generally dominated by sandy substrates with the occasional presence of rocky outcrops, boulders, woody debris, and other likely fish habitat. Examples of preferred fish habitat types observed at impacted sites in August 2018 are presented in Supplemental Data Figures SD3 to SD6.

Differences between sites in terms of their habitat attributes from the August 2018 survey were investigated using nonmetric multidimensional scaling (nMDS) plots (Euclidian distance matrices calculated on normalized data) for reservoirs and river sites. Some noticeable grouping of sites indicated similarities between habitat features observed at reservoirs and river sites, respectively (Supplemental Data Figure SD7), and the statistical analysis indicated that differences between sites were not significant (1‐way permutational analysis of variance [PERMANOVA], Reservoir sites: pseudo‐*F* = 0.66, *p* = 0.76; River sites: pseudo‐*F* = 1.69, *p* = 0.07). Although not significant, the following observations from these plots were made:

Reservoir sites

- Fumaca appeared to differ from other reservoir sites; this was due to less sand and more boulders and woody debris compared with other sites.
- Most of the other reservoir sites displayed a similar suite of attributes, except Baguari where boulders, woody debris, or fish nests were not observed.

River sites

- The 2 control sites Rio Piranga and Santo Antonio at Naque appeared to differ greatly from all other sites, indicating that the control site located downstream of the UHE Porto Estrela was more comparable to other impacted sites in terms of its habitat attributes.
- The apparent difference between Rio Doce at Naque and Rio Doce at BR262 compared with all other sites was likely associated with less diversity of the habitat attributes observed there, in particular little to no rocky structure.

## DISCUSSION

The acoustic survey program has generated a temporal biomass data set to track the recovery of aquatic habitats and fish stocks. The methodology is nondestructive and did not impose additional impacts on a potentially recovering ecosystem. A major benefit of the acoustic survey method was that it is not affected by turbid water and therefore can be used in both impact and control locations. High‐resolution sonar imaging allowed identification of underwater habitats down to individual woody snags and fish nests. Several methods implemented or developed for the present survey are proving to be useful for ongoing monitoring and establishing quantitative or semiquantitative targets for recovery of impact river locations.

Trends observed to date are limited in time. Follow‐up surveys will be key to the building of a comprehensive data set providing relevant evidence for interpretation. The results to date indicate that there is no pattern of relative fish biomass density that is consistent with a tailings effect at the sites investigated. Although the upper catchment ecology program (Hydrobiology 2020) reported a significant effect at impacted sites sampled in the Rio Gualaxo do Norte, it is important to note that the hydroacoustic program did not visit the same sites in the upper reaches and concentrated only on impoundments, which were not included in the upper ecology program.

Findings of the hydroacoustic program are based on comparisons between impact sites and available control sites, as well as preliminary temporal analyses to date. Fish biomass had not been investigated at these sites prior to the dam breach. Unfortunately, this program commenced a year and a half after the dam failure; therefore it is possible that the greatest biomass recovery may have occurred in the early period, which was not monitored. Evidence of early recovery includes the presence of fish nests at Resplendor and Aimores (Supplemental Data Figure SD6), in the lower section of the Rio Doce, from the first survey in April/May 2017. Although the initial peak recovery may not have been captured in the present program, it is important to continue monitoring to identify ongoing patterns of long‐term recovery.

## SUPPLEMENTAL DATA

Biomass results from hydroacoustics assessment and percentage of habitat cover from sonar assessment and a document with additional figures and methodology.

**Figure SD1.** Boxplots of temporal measurements of biomass at river sites in the lower Rio Doce.

**Figure SD2.** Boxplot of temporal measurements of biomass at reservoir sites in the lower Rio Doce. (Supplemental Information for Rio Doce Acoustic Surveys)

**Figure SD3.** Evidence of diverse fish habitats available at Dique S4 (down imaging). (Supplemental Information for Rio Doce Acoustic Surveys)

**Figure SD4.** Typical habitat observed at BR262 (side‐scan imaging). (Supplemental Information for Rio Doce Acoustic Surveys)

**Figure SD5.** Rocky habitat observed at Mascarenhas (down imaging on left, side‐scan imaging on right). (Supplemental Information for Rio Doce Acoustic Surveys)

**Figure SD6.** Fish nests observed at Aimores (side‐scan imaging). (Supplemental Information for Rio Doce Acoustic Surveys)

**Figure SD7.** nMDS plots of mean percentage benthic aquatic habitat coverage for reservoir (top) and river (bottom) sites during the August 2018 survey. (Supplemental Information for Rio Doce Acoustic Surveys)

**Table SD1.** Rio Doce catchment 2017–2018 hydroacoustic biomass data.

**Table SC2.** Rio Doce catchment August 2018 benthic habitat percentage data.

## Supporting information

This article contains online‐only Supplemental Data.

Supporting information

Supporting information

Supporting information

## Data Availability

Data used in this study are available in the Supplemental Data files.
