# Supplementary material for: Rio Doce Acoustic Surveys of Fish Biomass and Aquatic Habitat
Source: Integr Environ Assess Manag. 2020 Jun 18;16(5):615–21. doi: 10.1002/ieam.4285 (PMC7496619; doi:10.1002/ieam.4285)
Supplement: Supplementary file 3 — Supporting information [file IEAM-16-615-s003.docx]

Supplemental Data for

“**Rio Doce Acoustic surveys of Fish Biomass and Aquatic Habitat”**

Manuscript ID: IEAM-2019-200-SS

**Geographical coordinates of sites investigated**

| **Site name** | **River** | **Waterbody** | **Type** | **Latitude (WGS84)** | **Longitude (WGS84)** |
| --- | --- | --- | --- | --- | --- |
| PCH Fumaca | Rio Gualaxo do Sul | Reservoir | Control | -20.4540 | -43.2736 |
| UHE Brecha | Rio Piranga | Reservoir | Control | -20.5628 | -42.9701 |
| Dique S4 | Reservoir at Bento Rodrigues | Reservoir | Impact | -20.2402 | -43.4160 |
| RD at Candonga | Rio Doce | Reservoir | Impact | -20.2253 | -42.8773 |
| UHE Sa Carvalho | Rio Piracicaba | Reservoir | Control | -19.6486 | -42.8626 |
| UHE Porto Estrela | Rio Santo Antonio | Reservoir | Control | -19.1064 | -42.6627 |
| RD at Baguari | Rio Doce | Reservoir | Impact | -19.0291 | -42.1288 |
| Corrente | Rio Corrente | Reservoir | Control | -19.0349 | -42.1561 |
| RD at Aimores | Rio Doce | Reservoir | Impact | -19.4357 | -41.103 |
| RD at Mascarenhas | Rio Doce | Reservoir | Impact | -19.5083 | -41.0082 |
| Rio Piranga | Rio Piranga | River | Control | -20.3111 | -42.8963 |
| RD at BR262 | Rio Doce | River | Impact | -19.9793 | -42.6492 |
| RD at Ipatinga | Rio Doce | River | Impact | -19.4956 | -42.5184 |
| Rio Piracicaba | Rio Piracicaba | River | Control | -19.4928 | -42.5231 |
| RD at Perpetuo Socorro | Rio Doce | River | Impact | -19.3318 | -42.3696 |
| Santo Antonio at Naque | Rio Santo Antonio | River | Control | -19.2335 | -42.3379 |
| RD at Naque | Rio Doce | River | Impact | -19.2493 | -42.3131 |
| DS UHE Porto Estrela | Rio Santo Antonio | River | Control | -19.1267 | -42.6617 |
| RD at Resplendor | Rio Doce | River | Impact | -19.3517 | -41.2229 |
| Manhuacu | Rio Manhuacu | River | Control | -19.4896 | -41.1323 |
| RD at Itapina | Rio Doce | River | Impact | -19.5317 | -40.8083 |
| RD at Regencia | Rio Doce | River | Impact | -19.6503 | -39.8173 |

**Monitoring program site map**


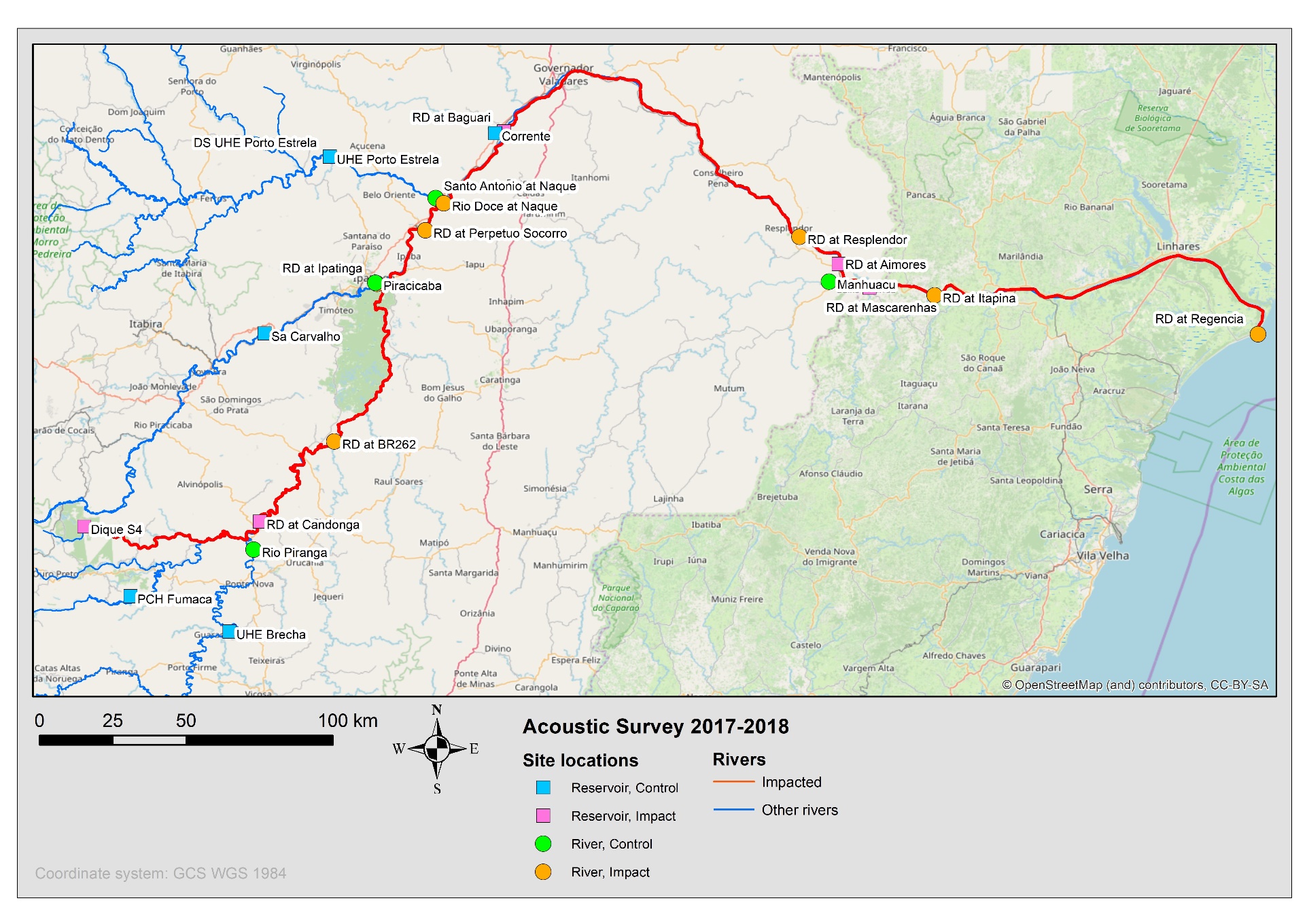


**Detailed hydroacoustic methods**

The mobile hydroacoustic surveys were conducted using a Biosonics DT-X echosounder with a 6.4° split beam transducer operating at 201 kHz, calibrated at the start of each survey using a Tungsten calibration sphere. The transducer was positioned facing downwards (or slightly angled forward depending on the bathymetry of the area) and the top was approximately 0.3 m below the water surface. Acoustic pulses were transmitted at a ping rate of 10 s^-1^, with a pulse length of 0.4 ms, and with a data collection threshold of -130 dB. Data were not collected in the first 0.99 m of the signal as this is the minimum transducer blanking distance. This means that the surveyed volume of water was between approximately 1.3 m below the surface and the bottom. The detection of fish in the water column was enumerated using echo detections which relate target strengths to biomass. The raw data were collected using the BioSonics Visual Acquisition software and georeferenced via connection to a Differential Global Positioning System (DGPS, Sokkia, <1cm accuracy).

Post survey analysis of acoustic echograms was undertaken using Sonar 5 Pro (Balk and Lindem, 2018) to enumerate biomass per unit volume of water. The algorithm used to estimate biomass values was a generic algorithm (referred to as ‘All_Species_1’ in v 6.0.4 of the software). This generic algorithm provides an appropriate estimate when dealing with mixed fish assemblages for which a site-specific algorithm has not been developed. Noise and interference were removed by using a minimum target threshold for processing of -65 dB. This threshold corresponds to fish of approximately 9 mm total length according to the equation of Love (1977), hence only fish greater than 9 mm were effectively captured in the data. Based on other hydroacoustic surveys in Minas Gerais, it is expected that this minimum threshold would also include swimming invertebrates such as, for example, *Chaoborus* (Bezerra-Neto *et al.*, 2012). Further processing was also performed manually to remove backscatter and other non-fish noise (e.g. aquatic vegetation, debris) on the echograms. The calculated biomass densities were exported to Excel and biomass values converted to a volume measure using the average fish weight and estimated volume of water analysed.

Statistical analyses of the biomass dataset were used to identify any differences between impacted versus control sites. Analyses of variance (ANOVA) were used to identify any treatment (impact/control) effect within a survey date. For temporal analyses, treatment (impact/control) effect was tested with consideration of other factors (survey date and/or seasons) using analyses of covariance (ANCOVA). A p-value below 0.05 indicated statistical significance.

**Additional Figures**


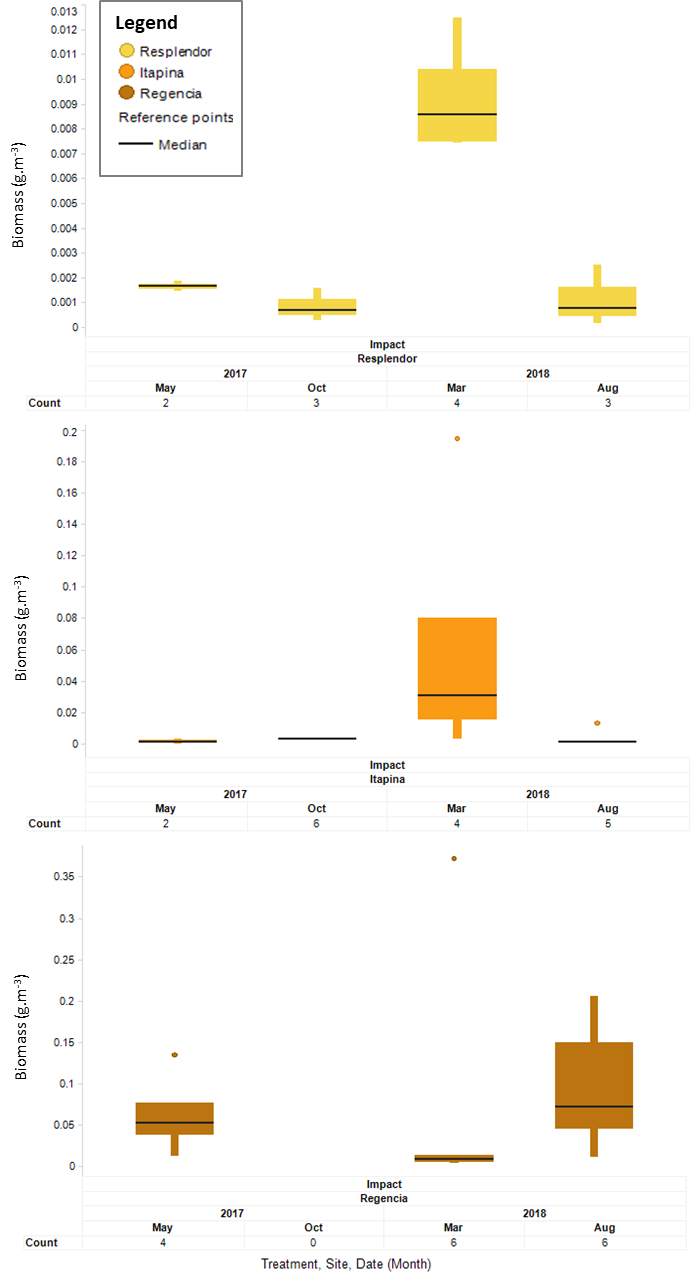


Figure SD 1 Boxplots of temporal measurements of biomass at river sites in the lower Rio Doce.


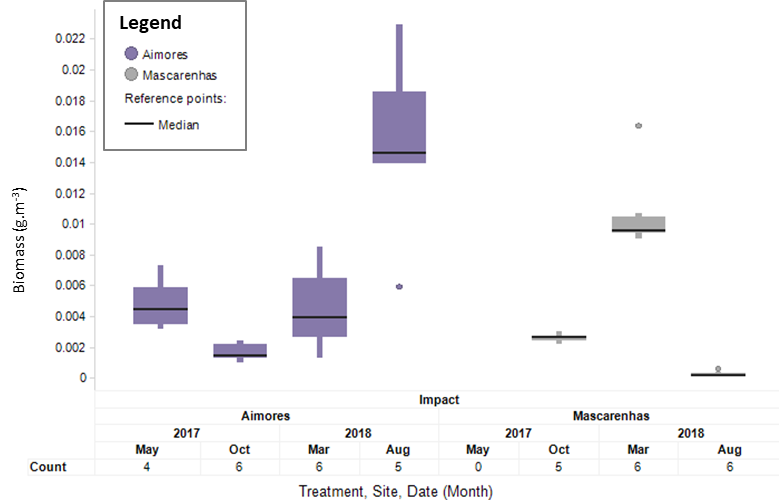


Figure SD 2 Boxplot of temporal measurements of biomass at reservoir sites in the lower Rio Doce.


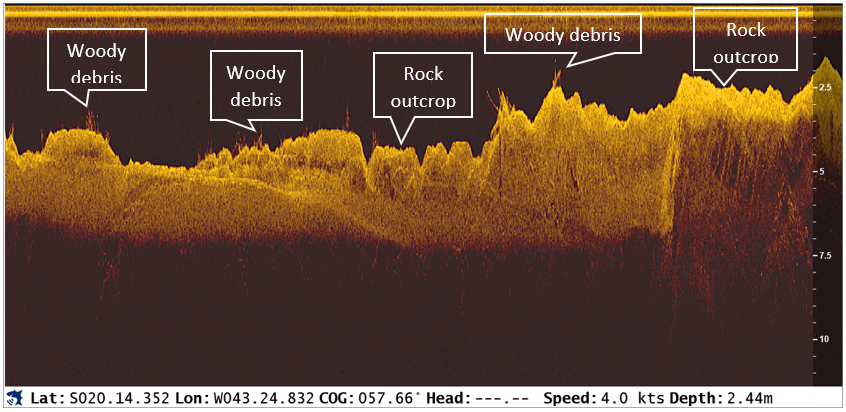


Figure SD 3 Evidence of diverse fish habitats available at Dique S4 (Down imaging)


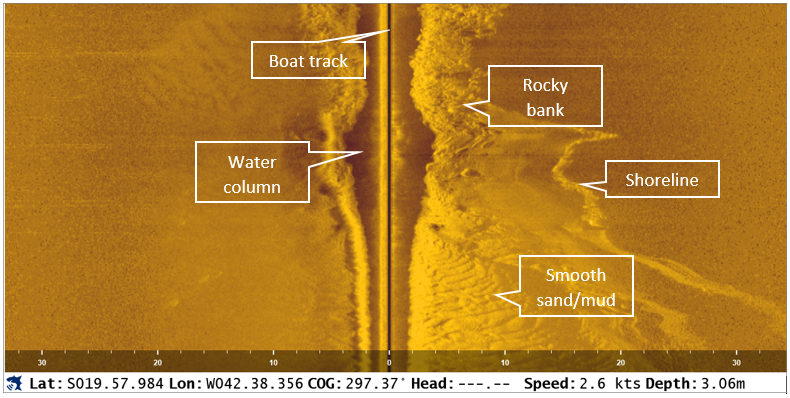


Figure SD 4 Typical habitat observed at BR262 (Side-scan imaging)


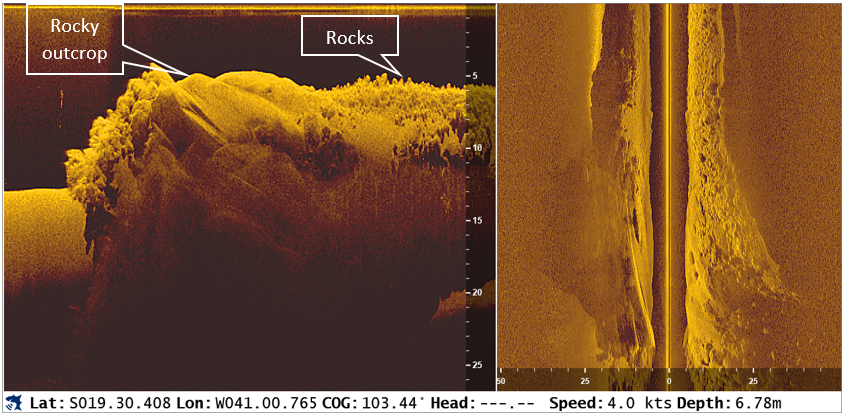


Figure SD 5 Rocky habitat observed at Mascarenhas (down imaging on left, side -scan imaging on right)


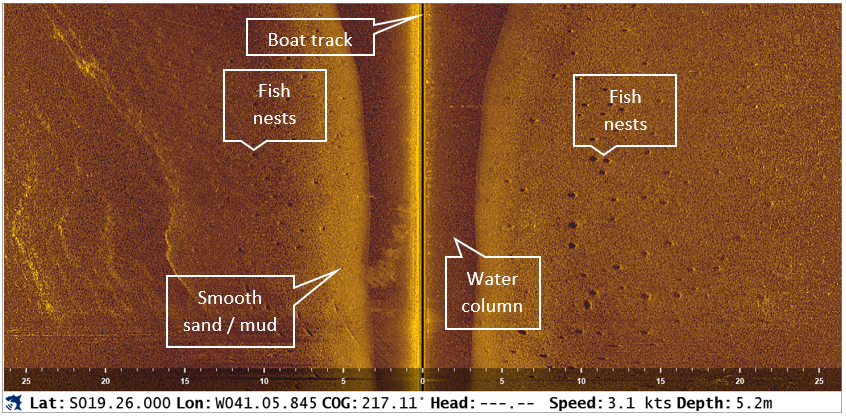


Figure SD 6 Fish nests observed at Aimores (side-scan imaging)


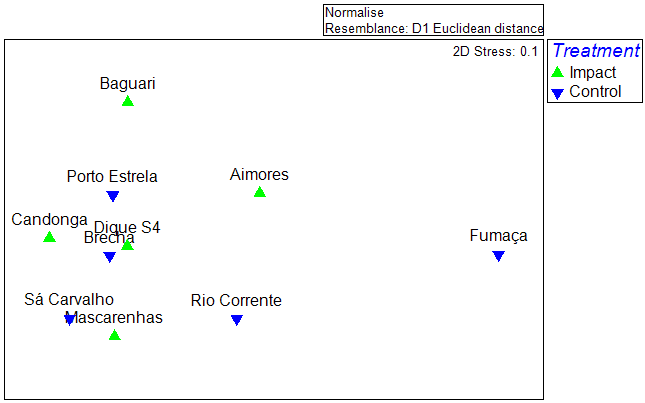


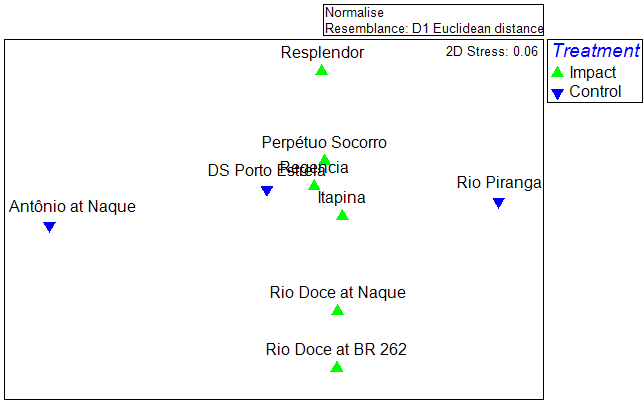


Figure SD 7 nMDS plots of mean percentage benthic aquatic habitat coverage for reservoir (top) and river (bottom) sites during the August 2018 survey.

Note: An MDS ordination attempts to represent the relative distance apart of different samples using a given number of dimensions, usually 2-D. The further apart a sample is represented, the less similar they are (Field *et al.*, 1982; Clarke and Ainsworth, 1993).

**References**

Balk, J. and Lindem, T. (2018) ‘Sonar 4 and Sonar 5 Pro: Post Processing System: Operator Manual Version 6.0.4.’

Bezerra-Neto, J. F., Brighenti, L. S., Mello, N. A. S. T. de and Pinto-Coelho, R. M. (2012) ‘Avaliação hidroacústica da distribuição de peixes e Chaoborus sp.(Diptera-Chaoboridae) em três lagos neotropicais’, *Acta Limnologica Brasiliensia*. SciELO Brasil, 24(1), pp. 18–28.

Clarke, K. and Ainsworth, M. (1993) ‘A method of linking multivariate community structure to environmental variables.’, *Marine Ecology-Progress Series 92*, p. 205.

Field, J., Clarke, K. and Warwick, R. (1982) ‘A practical strategy for analysing multispecies distribution patterns.’, *Marine ecology progress series*, pp. 37–52.

Love, R. (1977) ‘Target strength of an individual fish at any aspect’, *The Journal of the Acoustical Society of America*, 62(6), p. 1397.
